# Viral escape in the CNS with multidrug-resistant HIV-1

**DOI:** 10.7448/IAS.17.4.19745

**Published:** 2014-11-02

**Authors:** Charles Béguelin, Miriam Vázquez, Manuel Bertschi, Sabine Yerly, Denise de Jong, Andri Rauch, Alexia Cusini

**Affiliations:** 1 Department of Infectious Diseases, University Hospital and University of Bern, Bern, Switzerland; 2 Department of Neurology, University Hospital and University of Bern, Bern, Switzerland; 3 Laboratory of Virology, Geneva University Hospital, Geneva, Switzerland; 4 Department of Neuropsychology, University Hospital and University of Bern, Bern, Switzerland

## Abstract

**Introduction:**

HIV-1 viral escape in the cerebrospinal fluid (CSF) despite viral suppression in plasma is rare [1,2]. We describe the case of a 50-year-old HIV-1 infected patient who was diagnosed with HIV-1 in 1995. Antiretroviral therapy (ART) was started in 1998 with a CD4 T cell count of 71 cells/ìL and HIV-viremia of 46,000 copies/mL. ART with zidovudine (AZT), lamivudine (3TC) and efavirenz achieved full viral suppression. After the patient had interrupted ART for two years, treatment was re-introduced with tenofovir (TDF), emtricitabin (FTC) and ritonavir boosted atazanavir (ATVr). This regimen suppressed HIV-1 in plasma for nine years and CD4 cells stabilized around 600 cells/ìL. Since July 2013, the patient complained about severe gait ataxia and decreased concentration.

**Materials and Methods:**

Additionally to a neurological examination, two lumbar punctures, a cerebral MRI and a neuropsycological test were performed. HIV-1 viral load in plasma and in CSF was quantified using Cobas TaqMan HIV-1 version 2.0 (Cobas Ampliprep, Roche diagnostic, Basel, Switzerland) with a detection limit of 20 copies/mL. Drug resistance mutations in HIV-1 reverse transcriptase and protease were evaluated using bulk sequencing.

**Results:**

The CSF in January 2014 showed a pleocytosis with 75 cells/ìL (100% mononuclear) and 1,184 HIV-1 RNA copies/mL, while HIV-1 in plasma was below 20 copies/mL. The resistance testing of the CSF-HIV-1 RNA showed two NRTI resistance-associated mutations (M184V and K65R) and one NNRTI resistance-associated mutation (K103N). The cerebral MRI showed increased signal on T2-weighted images in the subcortical and periventricular white matter, in the basal ganglia and thalamus. Four months after ART intensification with AZT, 3TC, boosted darunavir and raltegravir, the pleocytosis in CSF cell count normalized to 1 cell/ìL and HIV viral load was suppressed. The neurological symptoms improved; however, equilibrium disturbances and impaired memory persisted. The neuro-psychological evaluation confirmed neurocognitive impairments in executive functions, attention, working and nonverbal memory, speed of information processing, visuospatial abilities and motor skills.

**Conclusions:**

HIV-1 infected patients with neurological complaints prompt further investigations of the CSF including measurement of HIV viral load and genotypic resistance testing since isolated replication of HIV with drug resistant variants can rarely occur despite viral suppression in plasma. Optimizing ART by using drugs with improved CNS penetration may achieve viral suppression in CSF with improvement of neurological symptoms.
